# Dissimilar patterns of tumor-infiltrating immune cells at the invasive tumor front and tumor center are associated with response to neoadjuvant chemotherapy in primary breast cancer

**DOI:** 10.1186/s12885-019-5320-2

**Published:** 2019-02-04

**Authors:** Lisa König, Fabian D. Mairinger, Oliver Hoffmann, Ann-Kathrin Bittner, Kurt W. Schmid, Rainer Kimmig, Sabine Kasimir-Bauer, Agnes Bankfalvi

**Affiliations:** 1 Department of Gynecology and Obstetrics, University Hospital Essen, University of Duisburg-Essen, Hufelandstr. 55, 45147 Essen, Germany; 2 Institute for Pathology, University Hospital Essen, University of Duisburg-Essen, Hufelandstr. 55, 45147 Essen, Germany

**Keywords:** Breast cancer, Tumor-infiltrating lymphocytes, Tumor microenvironment, Neoadjuvant chemotherapy, Minimal residual disease, Disseminated tumor cells, T cells, B cells

## Abstract

**Background:**

Tumor-infiltrating lymphocytes (TILs) are described as an important immune modulator in the tumor microenvironment and are associated with breast cancer (BC) outcome. The spatial analysis of TILs and TIL subtype distribution at the invasive tumor front (ITF) and the tumor center (TC) might provide further insights into tumor progression.

**Methods:**

We analyzed core biopsies from 87 pre-therapeutic BC patients for total TILs and the following subtypes: CD3+, CD4+, CD8+, CD20+ and CD68+ cells in correlation to clinicopathological parameters and disseminated tumor cells (DTCs) in the bone marrow.

**Results:**

TILs and TIL subtypes showed significantly different spatial distribution among both tumor areas. TILs, especially CD3+ T cells were associated with the tumor status and tumor grading. BC patients responding to neoadjuvant chemotherapy had significantly more TILs and CD3+ T cells at the TC. The presence of DTCs after NACT was related to CD4+ infiltration at the TC.

**Conclusion:**

The dissimilar spatial association of TILs and TIL subtypes with clinicopathological parameters, NACT response and minimal residual disease underlines the necessity of detailed TIL analysis for a better understanding of immune modulatory processes.

**Electronic supplementary material:**

The online version of this article (10.1186/s12885-019-5320-2) contains supplementary material, which is available to authorized users.

## Background

Breast cancer (BC) is the most common malignant tumor and the second leading cause of cancer-related death in women worldwide [1]. Due to significant progress in early diagnosis and individualized treatment options, the clinical outcome of BC has improved in the recent decades [2]. The main goals of neoadjuvant chemotherapy (NACT) are the reduction of tumor burden and monitoring tumor response to NACT [3]. However, a pathological complete response (pCR) after NACT can be achieved in 46 to 60% of the patients and 20% experience a metastatic relapse [4–6]. This might be explained by micrometastatic spread of tumor cells, preferentially to the bone marrow (BM) as disseminated tumor cells (DTCs) [7, 8]. The presence and persistence of these cells as well as their prognostic relevance has widely been shown [9–14] which indicates a rationale for testing alternative or secondary treatment options. In this regard, some efforts, including chemo-, antibody- or bisphosphonate therapy, have already been published [15–20].

There is evidence that inflammatory mediators released by cancer cells and tumor-infiltrating lymphocytes foster the development of a tumor microenvironment (TME) favoring tumor proliferation, migration, invasion as well as epithelial-mesenchymal transition (EMT) [21, 22]. Therefore, the composition and balance of anti- and pro-tumorigenic immune cells interspersing malignant tissue play a critical role for disease outcome [23]. Tumor-infiltrating immune cells are frequently observed in cancer, but the immune cell subtype composition differs among tumor entities [24]. The association of increased TILs with favorable prognosis in BC is widely accepted, however the role of different TIL subtypes remains controversial [25]. The predictive and prognostic significance of TILs was validated in large clinical studies, especially in triple negative (TNBC) and HER2 positive BC [26, 27]. Overall, a high density of TILs was described to be associated with a favorable outcome in NACT treated BC patients. However, subgroup analysis according to different molecular BC subtypes reported a correlation between high TILs, a poor outcome but a higher pCR rate [28, 29]. In a T cell subtype analysis, high levels of infiltrating CD8+ T cells were associated with a prolonged overall survival. CD4+ T helper cells were described to be a favorable prognostic factor with regard to overall clinical outcome [30, 31], while the influence of immunosuppressive regulatory CD4+ T cells still remains controversially debated [32–35]. B cells have been less extensively investigated, but are described to be associated with favorable disease outcome [36]. CD68+ macrophages were associated with worse prognostic features [37, 38] as well as shortened disease-free survival [38–40].

The crosstalk of tumor and immune cells located at the invasive tumor front (ITF) with the cells from the TME is essential for tumor progression and metastasis development [41, 42]. It has been demonstrated first in colorectal cancer (CRC) that CD3+ and CD8+ lymphocytes are spatially differentially distributed between the tumor center (TC) and the ITF and that the density and pattern of these immune infiltrates were associated with CRC patient outcome [43]. This led to the development of the so called Immunoscore assay to qualify and quantify the local host immune reaction to cancer cells, based on the numeration of two lymphocyte populations (CD3+ T cells and CD8+ cytotoxic T cells), both in the core of the tumor (TC) and in the invasive margin.

To the best of our knowledge, this is the first study analyzing the clinical impact of differential spatial distribution of TILs in primary BCs at baseline from NACT-treated patients in correlation to prognostic and predictive parameters as well as minimal residual disease. Here we evaluated densities and infiltration patterns of TILs and TIL subtypes separately in the TC and at the ITF from 87 pre-NACT BC core biopsies and correlated these findings to i) clinicopathological parameters, ii) response to NACT and iii) DTC status in the BM pre- and post-therapy.

## Material and methods

### Study design, patient and tumor characteristics

This retrospective, single-center study comprises 87 women with histologically proven primary invasive, non-metastatic BC who were diagnosed and treated between 2009 and 2012 at the Department for Gynecology and Obstetrics and the Institute of Pathology of the University Hospital Essen, Germany. Further eligibility criteria included: availability of bone marrow samples for DTCs at baseline and post-NACT, no severe co-morbidities, no further malignancies and surgical therapy after NACT with postoperative evaluation of the pathological response. Treatment decisions and follow-up were in accordance with international recommendations included in the German guidelines at that time. Indication for and composition of NACT regimens were described in detail previously [13]. The clinical response to NACT was graded on a three-tier scale as i) no change (pNC), ii) partial remission (pPR) and iii) pathologic complete remission (pCR) with no residual invasive or noninvasive tumor cells in breast and lymph nodes (ypT0,ypTis0, ypN0) [44]. Tumor type and stage were assessed according to the WHO-Classification of malignant tumors of the breast [45] and the sixth edition of the TNM Classification System [46]. The histopathological tumor grading pre-treatment was performed according to the Nottingham system elaborated by *Elston and Ellis* [47]. The histological regression grade post-NACT was determined using the four-tier classification of *Sinn* et al. [48]. Patient and tumor characteristics are listed in Table 1. All patients gave written informed consent for use of their tumor tissue for research purposes. The study was approved by the institutional ethics committee (16–6915-BO) and fully conforms to the principles outlined in the declaration of Helsinki.

**Table 1:** Patient and tumor characteristics

Total	87
Age	53 years (range: 28–83)
Menopausal status	
pre-menopausal	43 (50%)
peri-menopausal	6 (7%)
post-menopausal	38 (44%)
T stadium pre-NACT	
cT1a-cT1c	20 (23%)
cT2	51 (59%)
>cT2	14 (16%)
Unknown	2 (2%)
T stadium post-NACT	
ypT0, ypTis	18 (21%)
ypT1a	11 (13%)
ypT1b, ypT1c	18 (21%)
ypT2	26 (30%)
>ypT2	10 (12%)
Unknown	4 (5%)
N stadium pre-NACT	
cN0	41 (47%)
cN1	40 (46%)
cN2, cN3	5 (6%)
Unknown	1 (1%)
N stadium post-NACT	
yN0	47 (54%)
yN1	29 (33%)
yN2/N3	8 (9%)
Unknown	3 (4%)
Tumor type	
Ductal invasive	60 (69%)
Lobular invasive	11 (13%)
Other	14 (16%)
Unknown	2 (2%)
Tumor grade	
G1	4 (5%)
G2	40 (46%)
G3	42 (48%)
Unknown	1 (1%)
Estrogen receptor (IHC)^a^	
Positive	68 (78%)
Negative	19 (22%)
Progesterone receptor (IHC)^a^	
Positive	58 (67%)
Negative	29 (33%)
HER2 ^a^	
Positive	23 (26%)
Negative	64 (74%)
Tumor subtype	
ER-, PR-, HER2-	14 (16%)
ER-, PR-, HER2+	4 (5%)
ER+/PR+, HER2-	50 (58%)
ER+, PR+, HER2+	19 (22%)
NACT regimen	
CTX	53 (61%)
CTX + Trastuzumab	14 (16%)
CTX + Avastin	4 (5%)
CTX + Lapatinib + Trastuzumab	6 (7%)
HTX	9 (10%)
Unknown	1 (1%)
Trastuzumab treatment	
Yes	20 (23%)
No	66 (76%)
Unknown	1 (1%)
Clinical response	
pCR	15 (17%)
pPR	62 (71%)
pNC	6 (7%)
Unknown	4 (5%)
Histological tumor regression grade^b^	
0	6 (7%)
1	32 (37%)
2	22 (25%)
3	5 (6%)
4	12 (14%)
Unknown	10 (12%)
Local treatment	
Mastectomy	56 (64%)
Breast conservation surgery	26 (30%)
Unknown	5 (6%)
DTC positive	
pre-NACT	16 (18%)
post-NACT	13 (15%)

^a^Positive ER and PR status (ER+, PR+) was defined based on the guidelines of the American Society of Clinical Oncology [88] and concordant German recommendations at that time. Accordingly, we used a cut off value of 1% for ER and PR status. Breast cancers were defined as HER2-positive (HER2+) only for those cases that were + 3 by immunohistochemistry (IHC) (cut off 30%) or + 2 on IHC and confirmed positive by CISH [89]. Respective results were not re-defined because they served as a basis for therapy decision at baseline

^b^[48]

### Collection and analysis of DTCs from the bone marrow

BM was aspirated from the anterior iliac crests from BC patients prior to and after completing NACT (pre-NACT: *n* = 61, post-NACT: *n* = 72, matched samples: *n* = 47). All samples were obtained after written informed consent and collected using protocols approved by the institutional review board (05/2856). Isolation and detection of DTCs was performed according to current guidelines [49] and were described in detail elsewhere [13].

### Pathology assessment and immunohistochemistry

Routinely formalin-fixed and paraffin-embedded (FFPE) pre-NACT core biopsies were retrieved from the archives of the Institute of Pathology. An average number of 5 biopsies (range: 1 to 20) were procured per BC patient with a median size of 0.6 to 1.5 cm. Based on the availability of sufficient remaining tissue, core biopsies obtained before NACT were pre-screened on hematoxylin and eosin stained (H&E) sections for the presence of at least 30% tumor tissue and representative areas of TC and ITF within the biopsy. The TC was defined as intra-tumoral areas comprising malignant epithelial glands and desmoplastic tumor stroma with no direct connection to the peri-tumoral non-tumorus breast tissue. The ITF was restricted to a narrow band-like area at the tumor/host interface with a width of approximately 1 mm between the invasive edge of carcinoma tissue and the adjacent non-tumorus fibro-adipose stroma of the mammary gland [50]. The finally stained samples contained in average three biopsies (range: 1 to 4) per slide. In detail, one biopsy was on the slide in 10 cases, two biopsies in 34 cases, three biopsies in 37 cases and four biopsies in six cases. One slide per TIL subtype per patient (in total 6 slides per patients to evaluate) was stained as follows. Two μm thick serial tissue sections were cut and mounted on SuperFrost® Plus slides (Menzel, Braunschweig, Germany) for IHC. After deparaffinization and antigen retrieval (95 °C; 20 min citrate buffer), IHC was performed using the automated staining system BenchMark Ultra (Ventana Medical Systems, Tucson, USA) according to the manufacturer’s instruction. Staining was performed simultaneously on all slides with each antibody to avoid intersection variability. The following primary antibodies were used: CD3 (clone: SP7, DCS Innovative Diagnostik-Systeme, Hamburg, Germany; dilution: 1:400), CD4 (clone: 1F6, Zytomed Systems, Berlin, Germany; dilution: 1:40), CD8 (clone: C8/144B, DAKOCytomation, Glostrup, Denmark; dilution: 1:150), CD20 (clone: L26, DAKOCytomation, Glostrup, Denmark; ready to use), CD68 (clone: PG-M1, DAKOCytomation, Glostrup, Denmark; dilution: 1:500). Visualization of primary antibody binding was enabled using the OptiView DAB detection kit (Ventana Medical Systems, Tucson, USA). Positivity was defined by membranous staining of immune cells, irrespectively of the staining intensity. Human tonsil tissue sections served as routine positive control in each run and the staining quality was verified by the study breast pathologist.

### Assessment of tumor-infiltrating immune cells

One biopsy per patient was semi-quantitatively evaluated using a light microscope (Axioskop 2, Zeiss, Oberkochen, Germany) at 100x. The densities of the total TILs (H&E) and IHC-determined TIL subtypes were evaluated separately in the TC and at the ITF in the core biopsies, based on the recommendations of the *International TILs Working Group* and adapted with some practical modifications [23, 51]. Scores were defined as the percent proportion of the area infiltrated by the immune cells in the TC and at the ITF according to recommendations of *Denkert* et al. [28] and *Salgado* et al. [52], irrespective of intraepithelial or stromal localization. The quantity of TILs infiltration was classified into three categories: low (L) = 0–10%, moderate (M) = 11–30% and high (H) ≥ 31% infiltration. For binary statistics, low infiltrations were defined as “TIL-poor”, while moderate and high TILs were combined into “TIL-rich” categories.

In accordance with the *Immunoscore* (IS)-concept of *Galon* et al. [43, 53], two-marker immune profile estimations were also performed in situ by assessing the relative density of CD3+/CD8+, CD4+/CD8+, CD68+/CD8+, CD3+/CD20+ and CD68+/CD20+ immune cell populations in the TC and ITF regions [38, 54]. The dual scores were graded as follows: IS-G1 [high densities of both markers in both localizations (4xH)], IS-G2 [heterogeneous densities (3xH, 2xH, 1xH) for both markers and localizations] and IS-G3 [low densities of both markers at both areas (4xL) [55].

**Fig. 1:**
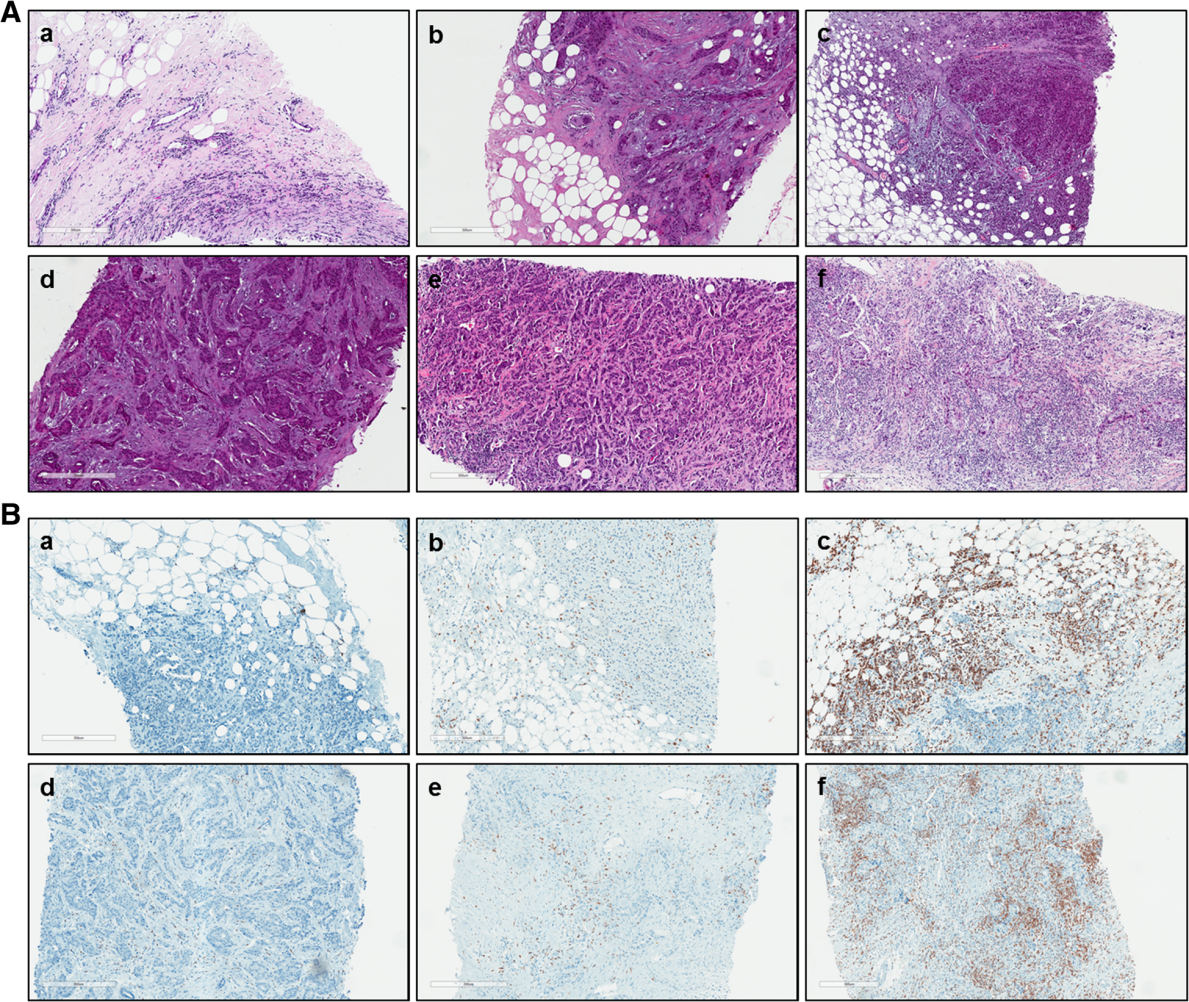
Representative H&E and immunohistochemistry (IHC) staining. Images show represenative H&E (**A**) and IHC staining for CD3 (**B**) at the ITF (**a-c**) and in the TC (**d-f**) for each category (**a/d** = Low, **b/e** = Moderate, **c/f** = High)

To minimize intra-observer variability, each staining was analyzed twice: (i) all slides from one immune cell subtype staining, (ii) all stained slides from one patient. The results were randomly re-checked by the study pathologist. A good inter-observer agreement was found (κ = 0,767). Figure 1 shows representative H&E staining (A) as well as CD3 IHC (B) for each infiltration category and for both tumor areas. IHC staining images for CD4, CD8, CD20 and CD68 are provided as supplementary material (Additional file 1: Figure S1).

### Statistical analysis

All statistical analyses were calculated using the R i386 statistical programming environment (v3.2.3). Before starting the literal statistical analysis, the Shapiro-Wilks-test was applied to test for normal distribution of the data. Subsequently, either a parametric or non-parametric test was applied. For dichotomous variables either the Wilcoxon Mann-Whitney rank sum test (non-parametric) or two-sided student’s t-test (parametric) was utilized. For ordinal variables with more than two groups, either the Kruskal-Wallis test (non-parametric) or ANOVA (parametric) was used to detect group differences. Double dichotomous contingency tables were analyzed using Fisher’s exact test. To test dependence to ranked parameters with more than two groups the Pearson’s Chi-squared test was applied. Correlations between metric or pseudometric parameters were tested by using the Pearson’s product moment correlation test for linear and the Spearman’s rank correlation test for non-linear regression, respectively. Due to the multiple statistical tests the *p*-values were adjusted by calculating the false discovery rate (FDR). Minimal sample size for each distinct group has been calculated using power calculation with Type I error probability of 0.05 and Type II error probability of 0.1. On a basis of a two-sample, two-sided t-test (SD = 1, delta = 1), the minimal sample size has been calculated as 23. The level of statistical significance was defined as *p* ≤ 0.05 after adjustment.

## Results

### Frequency distribution of total TILs and immune cell subtypes in pre-NACT core biopsies

**Fig. 2:**
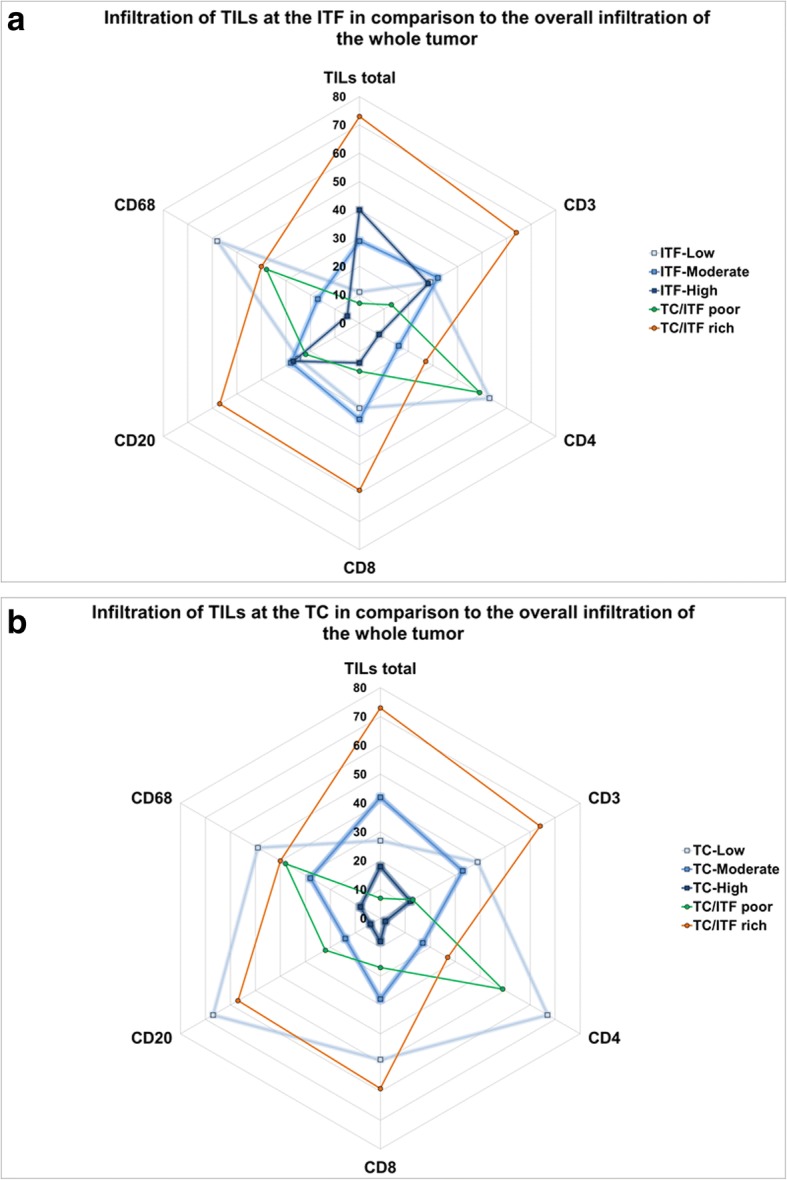
Distribution of TILs and TIL subtypes through the whole tumor. These radar plots show the number of breast carcinomas being infiltrated by the different TIL infiltration categories. TIL-poor tumors were defined as BCs with low level infiltration or complete lack of TILs both at the ITF and in the TC. TIL-rich tumors had a moderate or high level of immune cell infiltration at both intratumoral areas. **a** High levels of total TILs were mainly observed at the ITF, whereas the TC contained generally less immune cells. The ITF was most frequently interspearsed with CD3+ T lymphocytes, CD20+ B cells and CD8+ T cells. CD68+ macrophages and and CD4+ T cells cells were found in lower amounts at the ITF. **b** In the TC, CD3+ T cells were found to be the prevalent subtype, whereas low amounts of CD20+, CD68+, CD4+ and CD8+ cells were present, or there was no immune infiltration in the TC at all. The TIL infiltration is depicted by the number of tumors showing the certain infiltration densities

In total, 73 (84%) of the 87 tumors studied were infiltrated by TILs. The dominant TILs subtype across all BCs included CD3+ T lymphocytes, which were detected in 64 (74%) tumors, followed by CD8+ T cells in 59 (68%) and CD20+ B cells in 57 (66%) of the cases. Half of the tumors (50%) were infiltrated by CD68+ macrophages and 27 (31%) BCs had a CD4+ T cell infiltration (Fig. 2).

### Differential density distribution of total TILs and TIL subtypes at the ITF and in the TC

At the ITF, the majority of the tumors [*n* = 69, (80%)] were infiltrated at high and moderate levels by TILs. Regarding the quantity of infiltration by different cell types, high and moderate subtype density was observed most frequently for CD3+ lymphocytes [*n* = 60, (69%)], followed by CD20+ B cells in 55 (63%) and CD8+ lymphocytes in 48 cases (55%). CD68+ macrophages and CD4+ T cells were mainly present in low concentrations or lacked completely (Fig. 2a).

The TC was predominantly moderately infiltrated by TILs [*n* = 42, (48%)] and 27 (31%) of the tumors showed low level infiltration or were completely negative for TILs. 18 tumors (21%) were highly infiltrated by total TILs at the TC. CD3+ T cells represented the predominant subtype with high and moderate infiltration of the TC in 45 tumors (52%), while all other TIL subtypes were mainly present in low levels or lacked completely (Fig. 2b).

### Interrelationship between TILs and TIL subtype infiltration at the ITF and in the TC

Both intratumoral localizations showed a significant positive correlation between total TILs and immune cell subtype infiltrations. However, the composition of the immune cell infiltrate was significantly different in the two regions. At the ITF, the amount of total TILs was positively associated with all TIL subtypes, except CD68+ macrophages. In particular, the presence of CD3+ T cells was significantly correlated with CD4+ and CD8+ T lymphocytes (*p* = 5.35 E-5 and *p* = 0.0054) as well as with CD20+ B cell infiltrations (*p* = 1.39 E-5). The presence of CD20+ B cells was significantly related to infiltration with CD3+ T cells and CD8+ T lymphocytes (*p* = 1.39 E-5 and *p* = 0.0178). An additional association between CD8+ and CD4+ T cells was found (*p* = 0.0112). CD68+ macrophages were not associated with any TIL subgroup at the ITF (Table 2A).

**Table 2:** Correlation between TIL subtype infiltration at the TC and the ITF

**A: Correlation TILs ITF-ITF**							
**ITF**							
**ITF**		**TILs**	**CD3**	**CD20**	**CD68**	**CD4**	**CD8**
	**TILs**		**7.92 E-20** (0.83)	**3.50 E-7** (0.68)	0.2508 (0.32)	**0.0224** (0.54)	**0.0002** (0.51)
	**CD3**	**7.92 E-20** (0.83)		**1.39 E-5** (0.64)	1 (0.23)	**5.35 E-5** (0.63)	**0.0054** (0.57)
	**CD20**	**3.50 E-7** (0.68)	**1.39 E-5** (0.64)		1 (−0.02)	1 (0.26)	**0.0178** (0.40)
	**CD68**	0.2508 (0.32)	1 (0.23)	1 (−0.02)		0.3366 (0.32)	1 (0.01)
	**CD4**	**0.0224** (0.54)	**5.35 E-5** (0.63)	1 (0.26)	0.3366 (0.32)		**0.0112** (0.42)
	**CD8**	**0.0002** (0.51)	**0.0054** (0.57)	**0.0178** (0.40)	1 (0.01)	**0.0112** (0.42)	
**B: Correlation TILs TC-TC**							
**TC**							
**TC**		**TILs**	**CD3**	**CD20**	**CD68**	**CD4**	**CD8**
	**TILs**		**6.6 E-17** (0.78)	**0.0018** (0.44)	**0.0011** (0.45)	**0.0086** (0.4)	**5.08 E-5** (0.61)
	**CD3**	**6.6 E-17** (0.78)		**7.26 E-5** (0.5)	1 (0.1)	**0.0290** (0.37)	**2.05 E-7** (0.67)
	**CD20**	**0.0018** (0.44)	**7.26 E-5** (0.5)		1 (−0.02)	1 (0.21)	**0.0051** (0.55)
	**CD68**	**0.0011** (0.45)	1 (0.1)	1 (−0.02)		0.099 (0.34)	1 (0.1)
	**CD4**	**0.0086** (0.4)	**0.0290** (0.37)	1 (0.21)	0.099 (0.34)		0.0568 (0.36)
	**CD8**	**5.08 E-5** (0.61)	**2.05 E-7** (0.67)	**0.0051** (0.55)	1 (0.1)	0.0568 (0.36)	
**C: Correlation TILs ITF-TC**							
**ITF**							
**TC**		**TILs**	**CD3**	**CD20**	**CD68**	**CD4**	**CD8**
	**TILs**	**0.0005** (0.48)	**0.0003** (0.49)	**0.0251** (0.39)	0.924 (0.27)	**0.0023** (0.45)	0.1254 (0.35)
	**CD3**	**0.0013** (0.46)	**0.0005** (0.49)	**0.0013** (0.46)	1 (0.08)	**0.0011** (0.48)	**0.0086** (0.42)
	**CD20**	1 (0.12)	1 (0.08)	1 (0.23)	1 (−0.19)	1 (0.08)	1 (0.08)
	**CD68**	1 (0.22)	1 (0.23)	1 (0.03)	**0.0026** (0.45)	0.0653 (0.37)	1 (0.03)
	**CD4**	1 (0.1)	1 (0.16)	1 (0.05)	1 (0.25)	**0.0012** (0.47)	1 (0.12)
	**CD8**	1 (0.2)	1 (0.24)	0.495 (0.30)	1 (−0.09)	1 (0.21)	1 (0.2)

Bonferroni adjusted p-values (rho)

(A) ITF-ITF, (B) TC-TC and (C) ITF-TC. Results are given as Bonferroni adjusted p-values (rho). All bold values are significant p-values with "rho" in brackets

In the TC, total TILs and TIL subtypes were related to each other in a similar manner with some differences. Remarkably, CD68+ macrophages were here significantly associated with total TILs infiltration (*p* = 0.0011), whereas no correlation with T cell subtypes and B lymphocytes was found (Table 2B). Furthermore, CD 8+ T cells were not associated with CD4+ T cells in the TC.

The comparison of total TIL infiltration between the two areas revealed, if more total TILs were infiltrating the ITF, more were present in the TC and vice versa (*p* = 0.0005). Similarly, highly significant correlations were observed for CD3+ T cells (*p* = 0.0005), CD4+ T cells (*p* = 0.0012) and CD68+ macrophages (*p* = 0.0026) between the infiltration densities in the two different regions. The combined analysis of TC and ITF demonstrated a high proportion of total TILs in the TC significantly related to an elevated infiltration of CD3+ and CD4+ T cells (*p* = 0.0003 and *p* = 0.0023), as well as to CD20+ B cells at the ITF (*p* = 0.0251). A high amount of CD3+ cells at the TC was significantly related to high infiltrates of CD3+ (p = 0.0005), total TILs (*p* = 0.0013), CD4+ (*p* = 0.0011), CD20+ (*p* = 0.0013) and CD8+ cells (*p* = 0.0086) at the ITF. CD20+ B cells as well as CD8+ T lymphocytes differed between the two regions (Table 2C).

Overall, the composition of the immune infiltration was significantly different between the TC and the ITF regions. There was a highly significant accumulation of total TILs (*p* = 6.666 E-7) and all immune subsets at the ITF. Of note, CD20+ B cells were predominant at the ITF compared to the TC (*p* = 2.299 E-11). Higher densities of the T cell subsets with main subtypes of CD3+ (*p* = 1.109 E-6), CD8+ (*p* = 0.0031) and CD4+ T cells (*p* = 0.0073) were also present at the ITF. The difference for CD68+ macrophages was less significant (*p* = 0.0103) (data not shown).

### Association of TILs and TIL subtypes at the ITF and in the TC with clinicopathological parameters

Among the clinicopathological characteristics examined (Table 1), only tumor grade and tumor size pre- and post NACT were significantly associated with immune infiltrates in the pre-treatment core biopsies. The tumor grade was directly correlated with the degree of total TILs and CD3+ T cell infiltrations. Less differentiated tumors had significantly higher levels of TILs at both tumor areas (*p* = 0.047, respectively) and increased amount of CD3+ T cells at the ITF (*p* = 0.0407) (Table 3). Tumor size pre-NACT (cT-stage) was inversely correlated with the density of total TILs, CD3+ T cells and CD4+ T lymphocytes in the TC; they were highly present at early stages (cT1) and then decreased along with tumor progression (*p* = 0.004 for total TILs and CD3+ and *p* = 0.011 for CD4+ T cells). Tumor size post-NACT (ypT-stage) showed an inverse correlation with the degree of total TILs infiltration at the ITF (*p* = 0.0069) and in the TC (*p* = 0.0014) as well as with CD3+ T cells at the ITF (*p* = 0.001) and at the TC (p = 0.0069). Likewise, high levels of CD4+ and CD8+ T cells as well as CD20+ B cells at the ITF were significantly related to smaller tumors post-NACT (Table 3). An inverse correlation between post-NACT tumor stage and the grade of the Immunoscore-like dual marker combinations of CD3/CD8 and CD3/CD20 (*p* = 0.0254 and *p* = 0.0144) were observed, indicating that higher level infiltration of CD3+ T cells either with CD8+ T cells or with CD20+ B cells in both tumor regions was associated with a reduced tumor size after therapy.

**Table 3:** Association of TILs and TIL subtypes with clinicopathological characteristics

		p-value	
Clinical parameter	TIL subtype	ITF	TC
Tumor grade	TILs	0.047^a^	0.047^a^
	CD3	0.0407^a^	
cT stadium (pre-NACT)	TILs		0.004^d^
	CD3		0.004^d^
	CD4		0.0118^b^
ypT stadium (post-NACT)	TILs	0.0069^c^	0.0014^c^
	CD3	0.0010^c^	0.0069^c^
	CD4	0.0014^c^	
	CD8	0.0197^c^	
	CD20	0.0151^c^	
	IS CD3/CD8	0.0254^c^	
	IS CD3/CD20	0.0144^c^	
clinical response	TILs		0.0204^b^
	CD3		0.0235^b^
histological regression grade	TILs		0.0007^c^
	CD3	0.0308^c^	0.0002^c^
	CD4	0.037^c^	
	CD8	0.0308^c^	0.0008^c^
	CD20	0.023^c^	0.0196^c^
	IS CD3/CD8	0.0093^c^	
	IS CD3/CD20	0.0481^c^	
DTC presence post-NACT	CD4		0.0296^c^

High amounts of total TILs and CD3+ T cells at the ITF were associated with smaller tumor sizes post-NACT. Comparable results were observed for both immune infiltrates in the TC. The tumor grading was directly correlated with the amount of total TILs both at the ITF and in the TC as well as with the level of CD3+ cells at the ITF. Favourable NACT responses, evaluated by pathological response rate and histological regression grade, were directly associated with significantly higher amounts of total TILs and CD3+ cells present in the TC. Elevated amounts of CD4+ cells in the TC were associated with DTC presence post-NACT

^a^LM

^b^Kruskal-Wallis test

^c^Spearman

^d^Wilcoxon rank sum test

### Association of TILs and TIL subtypes at the ITF and in the TC with response to NACT

Response to NACT was primarily evaluated with regard to the pathological response rate (pCR) and secondly to the histological regression grade [48]. An improved response rate to NACT was observed with increasing amounts of total TILs and CD3+ T cells in the TC (*p* = 0.0204 and *p* = 0.0235) (Table 3). Higher histological regression grades were significantly associated with increased total TILs in the TC (*p* = 0.0007) as well as with elevated levels of CD3+ and CD8 + T lymphocytes and CD20+ B cells at both intratumoral regions. CD4+ infiltrates were only associated with tumor shrinkage after therapy when localized at the ITF (Table 3). It is of note, that the IS-like dual marker combinations CD3/CD8 and CD3/CD20 were directly related to the histological regression grade (*p* = 0.0093 and *p* = 0.0481) post-NACT (Table 3). There were no significant associations between TILs and the different NACT regimens applied.

### Correlation of TILs with tumor cell dissemination into the bone marrow

Overall, 19 BC patients showed DTCs in the BM at one time point, while 28 patients did not have DTCs before and after NACT. In average, one DTC was present before (range: 1–14) and after (range: 1–7) NACT. While no association with DTC presence before NACT was found, the presence of DTCs post-NACT was significantly associated with a higher incidence of CD4+ T cells at the TC (*p* = 0.0296) (Table 3).

## Discussion

Understanding the cellular and molecular mechanisms of the tumor immune microenvironment is becoming increasingly important with the emerging role of immunotherapy in breast cancer [56].

In the present study, we confirmed that pre-treatment TIL infiltration pattern significantly differed between ITF and TC in primary BC.

In line with the most published studies, TIL infiltration was found in more than 80% of all tumors and the overall predominant TIL subtype included CD3+ T cells, with CD8+ T cells being the main subset. The infiltration of CD4+ T cells was lower in our patient cohort than in the study of *Garcia-Martinez* et al. [54]. CD68+ macrophages were present in about the half of all tumors which is in concordance with other studies [54, 57]. The heterogeneous spatial distribution of TILs and TIL subtypes between the ITF and the TC has also been reported by *Mani* et al. [58]. Likewise, they found CD3+ T cells as the predominant TIL subtype in both tumor areas. Our finding of a significant accumulation of total TILs and all immune cell subtypes at the ITF provides evidence that the ITF represents an immunological hot spot area of communication between tumor and host. This phenomenon was also described for CRC [43, 55, 59] as well as in other solid tumors, including breast cancer [24, 38, 60, 61].

The positive correlation between total TILs and especially CD3+ T cells at the ITF and TC confirmed the observed TIL infiltration pattern with T cells as the dominant subtype while CD20+ B cells were predominantly located at the ITF. The concept of a time sequenced infiltration, B cells following the primarily responding T cells, provides an explanation for the spatially different subtype presence [62].

The correlation between TILs and immune cell subtypes with regard to chemotherapy response and survival has been investigated in various studies [25, 28, 35, 63, 64], but the association with clinicopathological parameters remains controversial [27, 28, 54, 65, 66]. Significantly higher TILs and CD3+ levels at the ITF in less differentiated tumors confirmed previous findings of others [66]. In contrast to other groups, we observed an association of smaller tumors post-NACT with a higher infiltration level of pre-treatment TILs and CD3+ cells at the ITF [66]. This may be explained by chemotherapy triggering or enhancing an anti-tumor immune response, thus, contributing to tumor remission [67].

Regarding the relationship between immune cell infiltration and clinicopathological patients’ and tumor characteristics, we did not find any association with the hormone receptor status, BC subtypes and any other parameters tested, in contrast to other studies [27, 66]. These divergent results might be explained by our separate spatial analysis of total TILs and immune cell subtypes at the ITF and the TC, whereas all other groups assessed the entire tumor.

Enhanced TILs and CD3+ T cells levels were found to be associated with a higher rate of pCR in BC patients in several studies [25, 27, 28, 35, 54, 63]. Besides the significant role of CD3+, CD8+ and CD20+ lymphocytes at both intratumoral regions in pCR, our results revealed that the infiltration of the TC with total TILs and CD3+ T cells was also significantly associated with NACT response and the pathological regression score [48] and was also described for entire tumor analyses in other neoadjuvant BC TIL studies [28, 29, 54, 63, 68]. Further, neoadjuvant BC trials investigating TNBC and HER2 positive BC subgroups [GeparQuattro [27], GeparQuinto [69], GeparSixto [70] and NeoALLTO [52] trials] confirmed this finding. This underlines again the clinical significance of the heterogeneous spatial distribution of TILs. In comparison to these trials we did not observe a relation of TILs to different NACT regimen [28, 69].

One of the novelties of the current study was the in situ analysis of different IS-like dual marker combinations. We demonstrated that higher CD3/CD8 and CD3/CD20 scores significantly correlated with advanced histological regression grades and reduced tumor size post-NACT. These findings support the central role of cytotoxic CD8+ T lymphocytes within the CD3+ T cell subset and the potential regulatory role of CD20+ B lymphocytes on T cell function during tumor cell elimination [36].

The *International TILs working group* defined recommendations for TILs analysis and suggested the inclusion of the spatial differential analysis at TC and ITF [23, 51]. The good inter-observer agreement in the present study was in line with others and proved this TIL evaluation method as independent from the observer [71, 72]. Commonly, TILs are analyzed from pre-NACT core biopsies or primary tumor tissue evaluating hematoxylin and eosin stained sections (H&E) and TIL subtypes were assessed by using immunohistochemical staining or immune gene signatures [25]. It has been demonstrated that the TIL distribution in a single biopsy of a tumor is representative of the whole tumor [58]. We are aware of the limitations of our study, as we were retrospectively analyzing a small cohort of early primary breast carcinomas. Besides described BC specific eligibility criteria, the availability of DTC analysis was mandatory and the investigation of the correlation to TILs was a main aim in this study. However, these stringent study criteria did not allow a differentiated BC subgroup analysis, which was a primary aim in the BIG 02–98 and the FinHER trial. Currently, there is no standard TILs evaluation method defined and used throughout all studies, which might explain the diverse results between the huge numbers of studies.

To the best of our knowledge, we are the first group investigating DTCs, which reflect pre-treatment tumor cell dissemination into the BM and minimal residual disease after NACT, in correlation with the cellular immunity in the tumor microenvironment. The presence of DTCs after NACT was significantly associated with higher levels of CD4+ T cells located in the TC pre-NACT. In this context, *DeNardo* et al. demonstrated metastatic spread into the lungs upon CD4+ T cells regulating pro-tumorigenic macrophage activity. However, a high CD4+/CD8+ ratio was not related to tumor cell dissemination in our study [73]. Regulatory CD4+ T cells can prevent the differentiation and expansion of cytotoxic T cells as well as DC maturation [74–76] and therefore prevent tumor elimination. The initiation of the pre-metastatic niche, e.g. in the BM, can be realized by CD4+ Tregs as well [77]. Remarkably, the CD4+ T cells in the TC, but not at the ITF correlated with DTCs. This leads to the suggestion that tumor cell dissemination may not depend on a direct physical interaction at the tumor-host interface, but rather on a communication via secreted cytokines/chemokines. It is conceivable, that CD4+ T cells facilitate dissemination of tumor cells exhibiting stem-cell character or an EMT phenotype. For BC, DTCs with stem cell character have already been detected [78, 79] and the molecular characterization of circulating tumor cells in this patient cohort identified tumor cells with a stem-cell phenotype or in EMT after the completion of NACT [13]. However, although the prognostic significance of DTCs has been demonstrated in a variety of studies, not all patients with detectable DTCs have a higher risk of relapse. The group by Falck et al., could not confirm the negative prognostic impact of DTCs in a cohort of 401 primary BC patients [80]. On the one hand, this might be explained by the use of immunofluorescence for staining of DTCS which is not the standard method that has been recommended after a consensus discussion [49]. Immunofluorescence is critical because of BM auto-fluorescence and endogenous immune cell properties as well as BM matrix components that can generate false-positive staining results which has been demonstrated very recently [81]. On the other hand, not all of the detected DTCs have metastatic potential. Pantel and Hayes, 2018, proposed an interesting model postulating that only patients who harbor DTCs with stem cell character with full metastatic potential of self-renewal and immortality among all other tumor bulk cells will develop metastasis years after first diagnosis [82]. Thus, before using DTCs as a diagnostic tool, a more precise characterization of the cells is necessary to identify patients who might have a higher risk for relapse.

BC was considered a non-immunogenic tumor entity before analyzing TIL infiltration [83] At current, the application of T cell-mediated immunotherapy of CTLA-4 [84, 85] and PD-L1 inhibitors [86] in combination with standard of care BC therapy in BC patients is quite promising [87]. However, the possibility of offering immunotherapies to BC patients depends on the tumor infiltration by certain immune cells. Our results contribute to increasing the knowledge about the complex interactions between BC and its inflammatory microenvironment with putative prognostic and predictive value.

## Conclusions

We demonstrated that dissimilar immune disposition at the ITF and TC is differentially associated with pro- and anti-tumor immunity, tumor cell dissemination and tumor response to contemporary NACT regimens in this cohort of early primary breast carcinomas. Further studies with comprehensive morpho-molecular evaluation of abundance, type and intra-tumoral location of tumor infiltrating immune cells might help to unravel their clinical relevance in the tumor immune microenvironment as well as their potential role as biomarker for or target of future treatment of breast cancer with personalized immuno therapies.

## Additional file

Additional file 1: **Figure S1.** Representative immunohistochemistry (IHC) staining images for CD8, CD4, CD20 and CD68. Images show representative images for CD8, CD4, CD20 and CD68 at the ITF (a-c) and in the TC (d-f) for each category (a/d = Low, b/e = Moderate, c/f = High). (TIF 2130 kb)

## Abbreviations

BC: Breast cancer
BM: Bone marrow
CD: Cluster of differentiation
CRC: Colorectal cancer
CTC: Circulating tumor cell
CTX: Chemotherapy
DTC: Disseminated tumor cell
ER: Estrogen receptor
FDR: False discovery rate
FFPE: Formalin-fixed paraffin-embedded
H: High
H&E: Hematoxylin and eosin
HER2: Human epidermal growth factor receptor-2
HTX: Hormonal therapy
IHC: Immunohistochemistry
IS: Immunoscore
ITF: Invasive tumor front
L: Low
M: Moderate
NACT: Neoadjuvant chemotherapy
pCR: Pathological complete response
pNC: No change
pPR: Partial response
PR: Progesterone receptor
TC: Tumor center
TIL: Tumor-infiltrating lymphocyte
TNBC: Triple negative breast cancer
