# Supplementary figures and images for: Dissimilar patterns of tumor-infiltrating immune cells at the invasive tumor front and tumor center are associated with response to neoadjuvant chemotherapy in primary breast cancer
Source: BMC Cancer. 2019 Feb 4;19:120. doi: 10.1186/s12885-019-5320-2 (PMC6360695; doi:10.1186/s12885-019-5320-2)

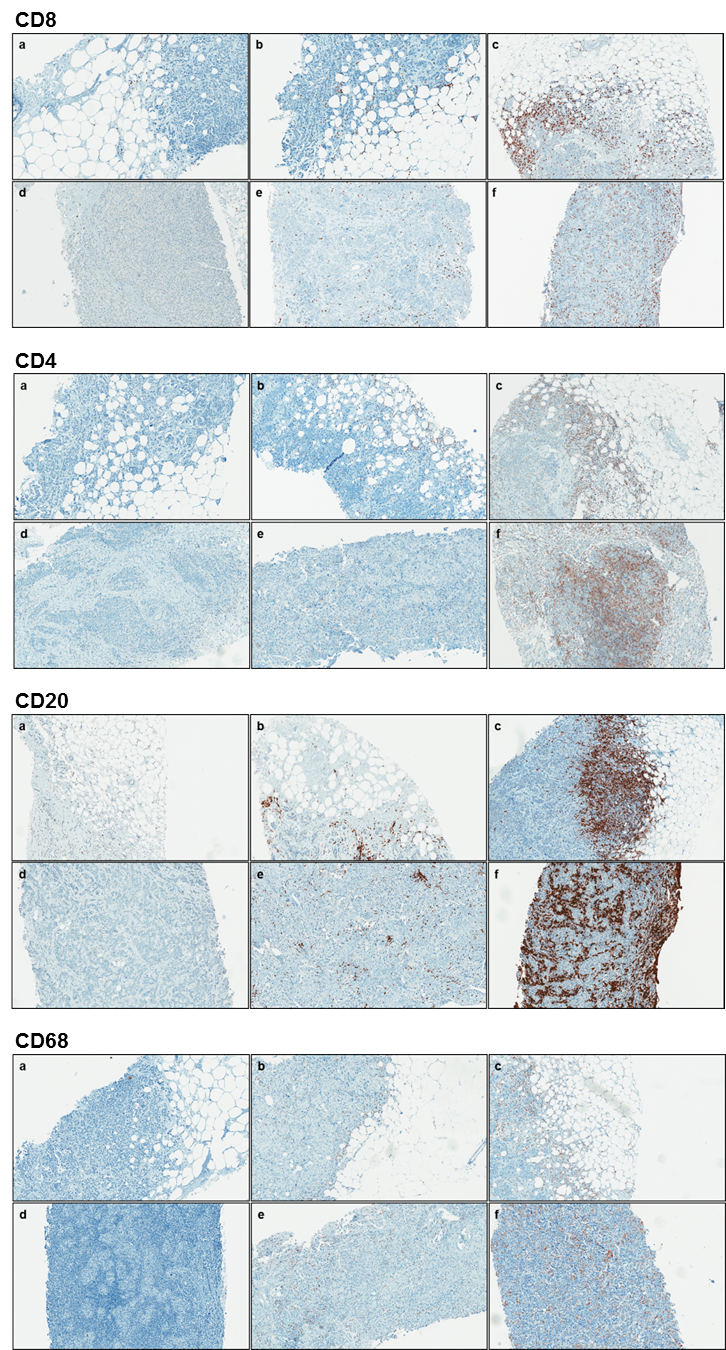

Supplement: Supplementary file 1 — Figure S1. Representative immunohistochemistry (IHC) staining images for CD8, CD4, CD20 and CD68. Images show representative images for CD8, CD4, CD20 and CD68 at the ITF (a-c) and in the TC (d-f) for each category (a/d = Low, b/e = Moderate, c/f = High). (TIF 2130 kb) [file 12885_2019_5320_MOESM1_ESM.tif]
